# Temporal trends of time to antiretroviral treatment initiation, interruption and modification: examination of patients diagnosed with advanced HIV in Australia

**DOI:** 10.7448/IAS.18.1.19463

**Published:** 2015-04-10

**Authors:** Stephen T Wright, Matthew G Law, David A Cooper, Phillip Keen, Ann McDonald, Melanie Middleton, Ian Woolley, Mark Kelly, Kathy Petoumenos, on behalf of the Australian HIV Observational Database

**Affiliations:** 1 The Kirby Institute, UNSW Australia, Sydney, Australia; 2 Health Protection, Communicable Diseases Branch, NSW Government Health, Sydney, Australia; 3 Department of Medicine, Monash University, Melbourne, Australia; 4 Department of Infectious Diseases, Monash University, Melbourne, Australia; 5 Infectious Diseases, Monash Medical Centre, Melbourne, Australia; 6 Brisbane Sexual Health and HIV Services, Brisbane, Australia

**Keywords:** combination antiretroviral therapy, late diagnosis, advanced HIV diagnosis, treatment interruption, treatment modification

## Abstract

**Introduction:**

HIV prevention strategies are moving towards reducing plasma HIV RNA viral load in all HIV-positive persons, including those undiagnosed, treatment naïve, on or off antiretroviral therapy. A proxy population for those undiagnosed are patients that present late to care with advanced HIV. The objectives of this analysis are to examine factors associated with patients presenting with advanced HIV, and establish rates of treatment interruption and modification after initiating ART.

**Methods:**

We deterministically linked records from the Australian HIV Observational Database to the Australian National HIV Registry to obtain information related to HIV diagnosis. Logistic regression was used to identify factors associated with advanced HIV diagnosis. We used survival methods to evaluate rates of ART initiation by diagnosis CD4 count strata and by calendar year of HIV diagnosis. Cox models were used to determine hazard of first ART treatment interruption (duration >30 days) and time to first major ART modification.

**Results:**

Factors associated (*p*<0.05) with increased odds of advanced HIV diagnosis were sex, older age, heterosexual mode of HIV exposure, born overseas and rural–regional care setting. Earlier initiation of ART occurred at higher rates in later periods (2007–2012) in all diagnosis CD4 count groups. We found an 83% (69, 91%) reduction in the hazard of first treatment interruption comparing 2007–2012 versus 1996–2001 (*p*<0.001), and no difference in ART modification for patients diagnosed with advanced HIV.

**Conclusions:**

Recent HIV diagnoses are initiating therapy earlier in all diagnosis CD4 cell count groups, potentially lowering community viral load compared to earlier time periods. We found a marked reduction in the hazard of first treatment interruption, and found no difference in rates of major modification to ART by HIV presentation status in recent periods.

## Introduction

Antiretroviral therapy (ART) is considered to be a life-long viable means to control HIV in patients and populations [1]. The introduction of fixed dose, once-a-day dosing and later generation antiretrovirals with more potency and fewer side effects has reduced treatment complexity, toxicity and ameliorated long-term adherence concerns. A developing body of evidence also suggests that initiating ART at higher CD4 cell counts translates into improved individual clinical outcomes [2], as well as a broader population-level reduction in community viral load (cVL) facilitating a reduction in HIV incidence [3–8]. In response, HIV prevention strategies are moving towards reducing plasma HIV RNA viral load in all HIV-positive persons, including those undiagnosed, treatment naïve, on or off ART.

In Australia, the rate of new HIV diagnoses has been steadily increasing since 1999 [9]. Of these newly diagnosed cases, the proportion presenting with later stage HIV disease has remained relatively stable over time [9]. Applying the European consensus definition for late presentation with HIV [10], approximately 40% of cases present late to care with CD4 cell count <350 cells/µL, and approximately 20% are diagnosed with advanced HIV disease (CD4 cell count <200 cells/µL). People with undiagnosed HIV for a large period of time might typically present late to care with advanced HIV and, by extension, might have been a higher risk of onwards transmission during their undiagnosed period contributing to an increasing incidence of HIV [11,12]. Identifying the characteristics of people who are at higher risk for presenting late to care with HIV and evaluating their shorter-term treatment experience might help inform clinical care practice for this large proportion of diagnoses.

The objectives of this analysis are to describe the population of patients presenting very late to care with HIV in Australia, identify patient characteristic correlates of these diagnoses, determine rates of antiretroviral therapy initiation from time of diagnosis and evaluate rates of treatment interruptions and major modifications to first-line ART.

## Methods

### Study population

The analysis population is based on patients recruited to the Australian HIV Observational Database (AHOD) by 31 March 2013. AHOD is an on-going longitudinal cohort study of HIV-positive patients in Australia. A comprehensive description has been previously described elsewhere [13,14]. Briefly, AHOD data are collected from 29 sites throughout Australia including hospital tertiary referral centres, sexual health clinics and general medical practices with a special interest in HIV. Written informed consent is obtained at time of cohort enrolment. The relevant Human Research Ethics Committees (HREC) has granted ethical approval for AHOD to all participating sites.

Data collection for AHOD commenced in 1999 and retrospective clinical data collected prior to the establishment of the cohort are provided where available. Data are transferred electronically to the Kirby Institute and subjected to quality control and quality assurance procedures. Data for AHOD are collected every six months on a core set of demographic and clinical variables, including sex, age, HIV exposure, hepatitis B virus surface antigen (HBsAg), hepatitis C virus antibody (HCV), CD4 and CD8 cell counts, plasma HIV viral load, antiretroviral treatment history, AIDS illnesses and date and cause of death.

### Data linkage

Date of HIV diagnosis and full CD4 cell count histories are collected in AHOD routinely where available. Due to recall bias and missing information, some of these data cannot be validated at the site data source level. To validate and further complete missing HIV diagnosis information in AHOD, data were linked to the Australian National HIV Registry (NHR) to obtain the date of the first HIV-positive test and corresponding CD4 cell count. The NHR was established in 1990 and incorporates all Australian state and territory health department records of HIV diagnoses from 1985 when HIV antibody testing first became available in Australia. A positive HIV antibody result is a notifiable event and requires reporting to state/territory health authorities. Information collected for each notification includes basic demographic and clinical characteristics [15]. The databases were linked using a two-pass, deterministic, one-to-many join (AHOD-to-NHR) data linkage procedure. The first pass joined the databases using an exact match on a unique identifier consisting of the concatenation of state/territory, sex, name code and date of birth information. The second pass took those not matched from pass one and removed the state/territory identifier from the linkage-key identifier. After pass two, any unmatched records were excluded from the analysis.

Approval to link AHOD and NHR was obtained by all Australian jurisdictional health departments and was reviewed and approved for ethical considerations by the New South Wales Population and Health Services Research Ethics Committee.

### Definitions & outcomes

To determine HIV presentation status, each participant had to have recorded a CD4 cell count at HIV diagnosis in the NHR. We defined presentation with advanced HIV disease as diagnosis with CD4 cell count <200 cells/µL or AIDS illness within six months of HIV diagnosis. We restricted our analysis population to AHOD participants who were diagnosed with HIV in the combination antiretroviral therapy era, defined as HIV diagnosis made after the 1 January 1996.

Initiation of first-line ART was the first recorded instance of the concurrent usage of ≥3 antiretrovirals for a duration >7 days. Of those participants who initiated ART, we established a broad timing of initiation group based on CD4 cell count at the time of diagnosis and CD4 at the time of ART initiation. Here, we are comparing treatment-related outcomes of those diagnosed with advanced HIV against those diagnosed with a higher CD4 cell count. We further refined the comparator arm by dichotomizing on CD4 cell count at the time of ART initiation. We categorized each participant into one of three groups,

1. Advanced HIV diagnosis: CD4 cell count at HIV diagnosis <200 cells/µL or AIDS illness within six months of diagnosis.
2. CD4_ART_ <350 cells/µL: CD4 cell count at HIV diagnosis >350 cells/µL and CD4 cell count at ART initiation <350 cells/µL.
3. CD4_ART_ ≥350 cells/µL: CD4 cell count at HIV diagnosis >350 cells/µL and CD4 cell count at ART initiation ≥350 cells/µL.

We defined the time to first treatment interruption as the first instance of ceasing ART continuously for >30 days. We defined the time to first major modification of ART as the removal, addition or switch of an antiretroviral class from the original ART regimen. Within class substitutions were not counted in our definition of a major ART modification.

For analyses examining time to first treatment interruption or first treatment modification, we wanted to directly compare treatment-related outcomes in non-late HIV diagnoses. Therefore, using the above definition, AHOD participants who were diagnosed with a CD4 cell count 200–350 cells/µL were excluded from the main analysis (*n*=179). However, as a sensitivity analysis, we varied the definition to capture theses late presenters, defined as diagnosis CD4 cell count <350 cells/µL or AIDS illness with six months of diagnosis.

### Statistical analysis

We tabulated patient characteristics stratified by CD4 cell count at time of HIV diagnosis (0–200, 201–350, >350 cells/µL). We assessed factors associated with advanced HIV diagnosis using logistic regression. We determined an adjusted odds ratio (aOR) for each factor using an *a priori* model adjusting for sex, age, mode of exposure, continental region of birth, hepatitis B co-infection, hepatitis C co-infection, primary care setting, regional care setting and year of HIV diagnosis. We assessed the statistical significance of each factor using a Wald test.

We used Kaplan–Meier survival methods to evaluate differences in rates of ART initiation stratified by CD4 cell at diagnosis (0–200, 201–350, 351–500, >500 cells/µL). To evaluate recent trends in ART initiation by CD4 cell count at diagnosis, we used an adjusted Cox proportional hazards model to formally test the interaction between calendar year of HIV diagnosis (groups: 1996–2000, 2001–2006, 2007–2012) and CD4 cell count at HIV diagnosis. The calendar year periods were broadly selected based on the availability of newer generation antiretrovirals (e.g. atripla) and alongside major changes in HIV treatment management guidelines (e.g. CD4 initiation threshold moving to <250 cells/µL). Further details on the evolution of the Australian HIV treatment guidelines can be found in Hoy and Lewin [16].

We used Cox proportional hazard models to evaluate HIV presentation status on the time to first ART interruption (>30 days) and the time to first major modification of ART. All models were adjusted for age, sex, mode of HIV exposure and primary care setting. Patients were considered lost-to-follow-up if they had not been seen at the clinic for >12 months. We censored patients at their last follow-up visit or at the administrative date of five years duration of ART, whichever occurred first.

Statistical significance was assessed at the *α*-level=0.05 level unless otherwise stated. All statistical calculations were performed using SAS Version 9.3.

## Results

A total of 2982 (83%) out of 3608 unique AHOD patient identifiers were matched to records from the NHR. Of the linked records, a total of 1599 were diagnosed HIV-positive after 1 January 1996. A further 397 participants were excluded from the analysis due to missing diagnosis CD4 cell count. A grand total of 1202 were eligible for analysis. The linked NHR diagnosis date is more specific and an improvement on corresponding AHOD records as shown by comparing the frequency distribution of each component of the HIV diagnosis date (day, month, year), Supplementary Figure 1. A total of 528 (43%) diagnosis CD4 cell count records were completed (or gained) after the data linkage procedure. Supplementary Figure 2 demonstrates almost perfect concordance of available AHOD CD4 cell count data with those captured by the NHR. Patient characteristics of those linked/not-linked or included/excluded in the analysis are presented in Supplementary Table 1.

The proportion of participants in our analysis of population diagnosed with advanced HIV was 0.21 (0.18, 0.23). The proportion of participants diagnosed with late HIV was 0.35 (0.33, 0.38). Analysis of population-patient characteristics is shown in Table 1. Univariate and multivariable aORs for factors associated with advanced HIV diagnoses are presented in Table 2. Factors associated with diagnosis of advanced HIV include: sex, female aOR=0.50 (0.26, 0.98) relative to male; older age, >50 years aOR=4.91 (2.74, 8.76) relative to <30 years old; mode of HIV exposure, heterosexual aOR=2.04 (1.38, 3.02) or other (blood products, needle sticks, etc.) aOR=2.05 (1.08, 3.86) compared to men who have sex with men (MSM); born in high HIV-prevalence countries, Africa aOR=3.87 (1.49, 10.1) and Asia aOR=2.57 (1.44, 4.58) relative to those born in Australia; participants receiving care from a hospital tertiary referral clinic, aOR=2.88 (1.57, 5.30) compared to those receiving care at a sexual health clinic. We found no interactions between factors studied with year of diagnosis. This implies no large temporal changes in factors associated with advanced HIV diagnoses over time. Sensitivity analyses of aORs for late diagnosis are shown in Supplementary Table 2 and qualitatively similar results were found.

**Table 1:** Analysis of population-patient characteristics by CD4 cell count at diagnosis

		CD4 cell count at HIV diagnosis		
		
Factor	Level	CD4 <200 cells/µL *N* (%)	CD4 201–350 cells/µL *N* (%)	CD4 >350 cells/µL *N* (%)
Number of patients	(Total *n*=1202)	245 (20)	185 (15)	772 (65)
Year of cohort enrolment	1999–2004	135 (55)	82 (44)	280 (36)
	2005–2009	72 (29)	56 (30)	279 (36)
	2010–2012	38 (16)	47 (25)	213 (28)
Sex	Female	14 (6)	16 (9)	54 (7)
	Male	231 (94)	169 (91)	718 (93)
Age at cohort enrolment (years)	<30	18 (7)	29 (16)	135 (17)
	30–40	69 (28)	58 (31)	293 (38)
	40–50	68 (28)	59 (32)	232 (30)
	>50	90 (37)	39 (21)	112 (15)
Mode of probable HIV exposure	MSM	144 (59)	127 (69)	571 (74)
	Heterosexual	70 (28)	45 (24)	146 (19)
	IDU	12 (5)	5 (3)	30 (4)
	Other	19 (8)	8 (4)	25 (3)
Continental region of birth	Australia	135 (55)	110 (59)	502 (65)
	New Zealand/Pacific Islander	4 (2)	5 (3)	21 (3)
	Africa	9 (4)	6 (3)	8 (1)
	Americas	6 (2)	2 (1)	9 (1)
	Asia	22 (9)	14 (8)	42 (5)
	Europe	20 (8)	14 (8)	41 (5)
	Not reported	49 (20)	34 (18)	149 (19)
Hepatitis B SurAg (ever)	Positive	10 (4)	7 (4)	18 (2)
	Negative	199 (81)	148 (80)	598 (77)
	Not reported	36 (15)	30 (16)	156 (20)
Hepatitis C antibody (ever)	Positive	18 (7)	7 (4)	59 (8)
	Negative	211 (86)	165 (89)	634 (82)
	Not reported	16 (7)	13 (7)	79 (10)
Australian state capital city setting	Capital city	162 (66)	126 (68)	565 (73)
	Non-capital city	83 (34)	59 (32)	207 (27)
Primary care setting	General practice	76 (31)	64 (35)	327 (42)
	Hospital clinic	68 (28)	34 (18)	145 (19)
	Sexual health clinic	101 (41)	87 (47)	300 (39)

MSM=men who have sex with men; IDU=injecting drug use.

**Table 2:** Factors associated with advanced HIV diagnosis: defined by CD4 cell count at diagnosis <200 cells/µL and or new AIDS illness within six months of HIV diagnosis

		Univariate model		Multivariable model	
		
Factor	Level	OR (95% CI)	*P*^a^	aOR (95% CI)	*P*^a^
Sex	Female	0.841 (0.46, 1.45)	0.48	0.50 (0.26, 0.98)	0.04
	Male	1.00 (ref)		1.00 (ref)	
Age at diagnosis (years)	<30	1.00 (ref)	<0.01	1.00 (ref)	<0.01
	30–40	1.82 (1.05, 3.16)		1.73 (0.98, 3.05)	
	40–50	2.21 (1.27, 3.83)		2.07 (1.16, 3.68)	
	>50	5.73 (3.30, 9.94)		4.91 (2.74, 8.76)	
Mode of probable HIV exposure	MSM	1.00 (ref)	<0.01	1.00 (ref)	<0.01
	Heterosexual	1.79 (1.29, 2.47)		2.04 (1.38, 3.02)	
	IDU	1.61 (0.82, 3.17)		1.96 (0.92, 4.18)	
	Other	2.70 (1.49, 4.88)		2.05 (1.08, 3.86)	
Continental region of birth	Australia	1.00 (ref)	0.02	1.00 (ref)	<0.01
	New Zealand/Pacific Islander	0.69 (0.25, 2.09)		0.72 (0.22, 2.20)	
	Africa	2.89 (1.23, 6.81)		3.87 (1.49, 10.08)	
	Americas	3.15 (1.18, 8.41)		2.60 (0.88, 7.70)	
	Asia	1.88 (1.12, 3.16)		2.57 (1.44, 4.58)	
	Europe	1.75 (1.02, 2.99)		1.78 (0.98, 3.21)	
	Not reported	1.27 (0.88, 1.82)		0.79 (0.53, 1.20)	
Hepatitis B co-infection	Positive	1.45 (0.69, 3.08)	0.14	1.41 (0.64, 3.15)	0.67
	Negative	1.00 (ref)		1.00 (ref)	
	Not reported	0.73 (0.49, 1.07)		1.08 (0.64, 1.75)	
Hepatitis C co-infection	Positive	1.00 (0.58, 1.71)	0.27	0.99 (0.54, 1.81)	0.41
	Negative	1.00 (ref)		1.00 (ref)	
	Not reported	0.64 (0.37, 1.10)		0.62 (0.31, 1.25)	
Australian state capital city setting	Capital city	1.00 (ref)	0.02	1.00 (ref)	<0.01
	Non-capital city	1.43 (1.06, 1.92)		2.40 (1.34, 4.31)	
Primary care setting	General practice	0.69 (0.50, 0.96)	<0.01	1.36 (0.75, 2.45)	<0.01
	Hospital clinic	1.43 (1.00, 2.02)		2.88 (1.57, 5.30)	
	Sexual health clinic	1.00 (ref)		1.00 (ref)	
Year of HIV diagnosis	1996–2000	1.00 (ref)	<0.01	1.00 (ref)	<0.01
	2001–2006	0.46 (0.32, 0.65)		0.44 (0.31, 0.67)	
	2007–2012	0.72 (0.51, 1.02)		0.70 (0.48, 1.02)	

a Wald test for categorical level heterogeneity. MSM=men who have sex with men; IDU=injecting drug use.

**Figure 1:**
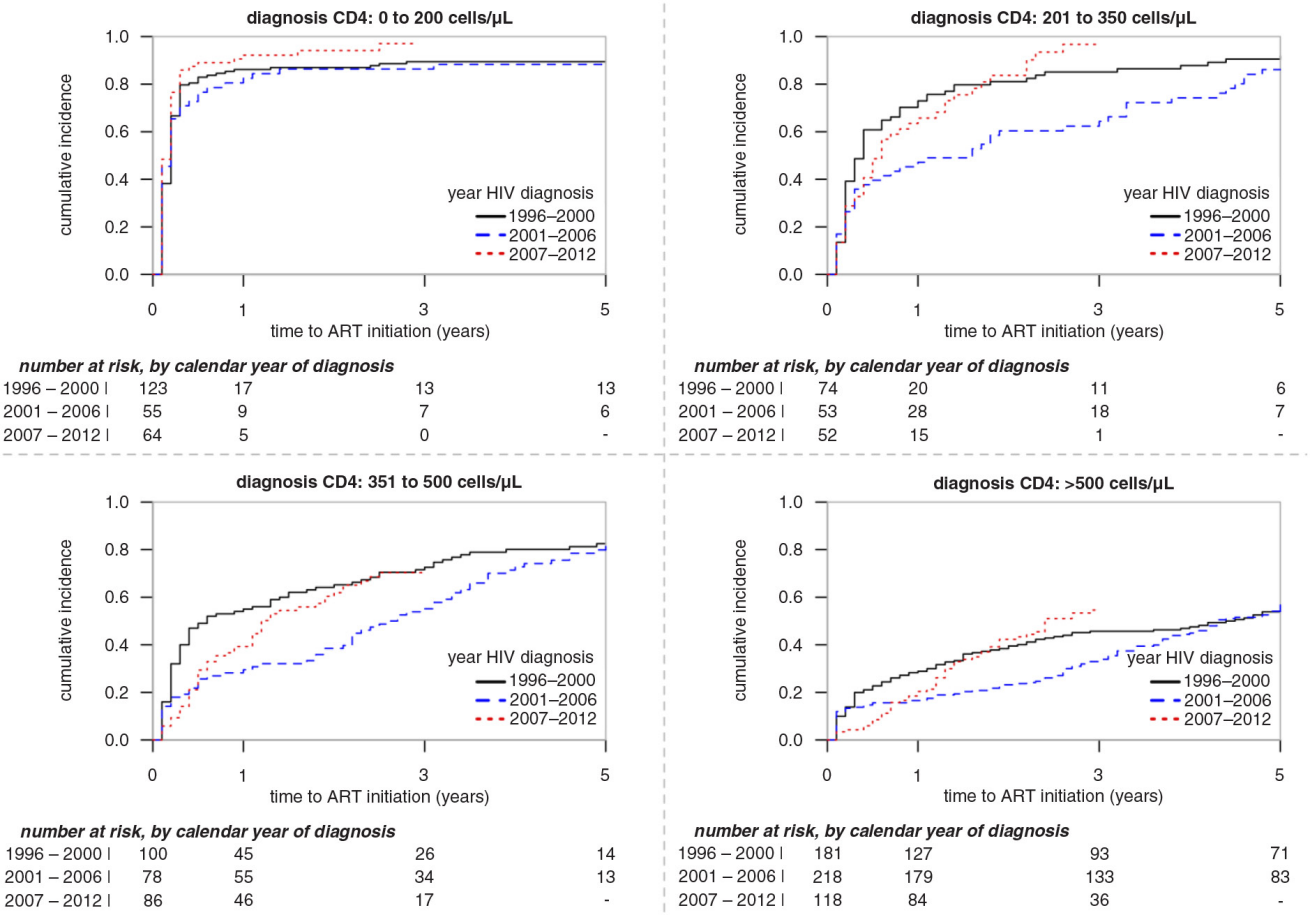
Time from first HIV-positive diagnosis to ART initiation stratified by year of diagnosis and CD4 cell count at HIV diagnosis.

Rates of ART initiation differed by diagnosis CD4 cell count strata, *p*<0.0001 and year of HIV diagnosis, *p*<0.0001 (Figure 1). The adjusted model interaction term between diagnosis CD4 cell count strata and year of HIV diagnosis was insignificant (*p*=0.08), indicating the ART initiation patterns for year of HIV diagnosis did not differ within each diagnosis CD4 cell count stratum (Figure 1). The majority of participants diagnosed with a CD4 count <500 cells/µL initiated ART within five years of their HIV diagnosis. For recent HIV diagnoses (2007–2012) and using Kaplan–Meier estimates, the time for 50% of participants initiating ART by diagnosis CD4 cell count strata were 1 (1, 2) month, 6 (4, 14) months, 14 (12, 24) months and 28 (23, 43) months for groups 0–200, 201–350, 351–500 and >500 cells/µL, respectively.

The estimated hazard ratios for time to first ART treatment interruption stratified by HIV presentation status are presented in Table 3. We found an 83% (69, 91%) reduction in the hazard of first treatment interruption in recent time periods (2007–2012) relative to earlier time periods (1996–2000), *p*<0.001. We demonstrate a higher hazard of treatment interruption in those who initiated ART therapy with a higher CD4 cell count aHR=1.96 (1.22, 3.20) compared to those that started with a lower count (CD4 < 350 cells/µL), *p*<0.001. We found no difference in hazard of treatment interruption in those diagnosed with advanced HIV (and initiated ART) compared to those who started with a CD4 cell count <350 cells/µL. An interaction term between HIV presentation status and calendar year was non-significant (*p*=0.93), indicating that broad patterns of treatment interruption hazards for HIV presentation status are similar within each time period. Similar results were found comparing late diagnosis to CD4_ART_ <350 cells/µL or CD4_ART_ ≥350 cells/µL groups (Supplementary Table 3).

**Table 3:** Cox proportional hazard^a^ of time to first ART treatment interruption (>30 days) by HIV presentation category

Factor	Interrupt/person-years (py)	Rate (per 100 py) (95% CI)	aHR^a^	*p*
Year of ART initiation				
1996–2000	83/784	10.6 (8.5, 13.0)	1.00 (ref)	<0.001
2001–2006	57/670	8.5 (6.5, 10.9)	1.05 (0.72, 1.53)	
2007–2012	13/741	1.8 (0.9, 3.0)	0.17 (0.09, 0.31)	
HIV presentation status^b^				
adv. HIV diagnosis	36/799	4.5 (3.2, 6.2)	0.86 (0.49, 1.50)	<0.001
CD4_ART_ <350 cells/µL	24/596	4.0 (2.6, 5.9)	1.00 (ref)	
CD4_ART_ ≥350 cells/µL	93/800	11.6 (9.5, 14.1)	1.96 (1.22, 3.20)	

a Model adjusted for year of ART initiation, HIV presentation status, age, sex, HIV exposure, primary care clinic type

b *adv. HIV diagnosis*: CD4 cell count at HIV diagnosis <200 cells/µL and or new AIDS illness within six months of diagnosis; *CD4*_*ART*_ *<350 cells/µL*: CD4 cell count at HIV diagnosis >350 cells/µL and CD4 cell count at ART initiation<350 cells/µL; *CD4*_*ART*_ *≥350 cells/µL*: CD4 cell count at HIV diagnosis>350 cells/µL and CD4 cell count at ART initiation ≥350 cells/µL.

The hazard of a first major modification to ART by HIV presentation status is presented in Table 4. An interaction term between HIV presentation status and recent time period was statistically significant, *p*=0.03. We found no differences in ART modification hazard for HIV presentation status in year of ART initiation groups 2001–2006 and 2007–2012. In the time period 1996–2000, the rate of modifying ART regimens in advanced HIV diagnosis and those that started ART with a CD4 ≥350 cells/µL were higher than those that initiated ART with a lower CD4 count, 4.13 (1.28, 13.34) and 2.46 (0.75, 8.07) respectively. Again, comparing late diagnosis to the other groups we found similar results (Supplementary Table 4).

**Table 4:** Cox proportional hazard^a^ of time to switch to second line, by HIV presentation status

Factor	Switch/person-years (py)	Rate/100 py (95% CI)	aHR^a^	*p*
Year of ART initiation: 1996–2000				
adv. HIV diagnosis^b^	46/317	14.5 (10.8, 18.9)	4.13 (1.28, 13.34)	0.01
CD4_ART_ <350 cells/µL	3/87	3.4 (0.7, 9.7)	1.00 (ref)	
CD4_ART_ ≥350 cells/µL	32/381	8.4 (5.8, 11.6)	2.46 (0.75, 8.07)	
Year of ART initiation: 2001–2006				
adv. HIV diagnosis	16/207	7.7 (4.5, 12.2)	0.73 (0.38, 1.40)	0.15
CD4_ART_ <350 cells/µL	22/204	10.1 (6.9, 15.9)	1.00 (ref)	
CD4_ART_ ≥350 cells/µL	16/314	5.1 (209, 8.1)	0.52 (0.27, 1.01)	
Year of ART initiation: 2007–2012				
adv. HIV diagnosis	13/167	7.8 (4.2, 12.9)	0.85 (0.43, 1.69)	0.22
CD4_ART_ <350 cells/µL	24/249	9.6 (6.3, 14.0)	1.00 (ref)	
CD4_ART_ ≥350 cells/µL	30/203	14.8 (10.1, 20.4)	1.43 (0.83, 2.45)	

a Model adjusted for year of ART initiation, HIV presentation status, age, sex, HIV exposure, primary care clinic type

b *adv. HIV diagnosis*: CD4 cell count at HIV diagnosis <200 cells/µL and or new AIDS illness within six months of diagnosis; *CD4*_*ART*_ *<350 cells/µL*: CD4 cell count at HIV diagnosis >350 cells/µL and CD4 cell count at ART initiation <350 cells/µL; *CD4*_*ART*_ *≥350 cells/µL*: CD4 cell count at HIV diagnosis >350 cells/µL and CD4 cell count at ART initiation ≥350 cells/µL.

## Discussion

In our study, we used a data linkage methodology to ascertain the date of HIV diagnosis and CD4 cell count at diagnosis for each participant in AHOD. We used these data to estimate the proportion of participants diagnosed with advanced HIV in AHOD and identify associations of increased odds of advanced HIV diagnosis. To our knowledge, for the first time using Australian data, we have characterized the rates of ART initiation from first HIV-positive test date by diagnosis CD4 cell count strata. Stratifying the analysis population by CD4 cell count at HIV diagnosis and year of HIV diagnosis, we were able to highlight the different approaches for the treatment of asymptomatic HIV-positive patients followed and characterize the impact on rates of ART treatment initiation. We have shown a large reduction in antiretroviral treatment interruptions in more recent time periods, as well as found no difference in the rate of major first-line ART modification in participants diagnosed with advanced HIV compared to those diagnosed with a CD4 cell count ≥350 cells/µL.

A major strength of this analysis is the use of data linkage methods. We matched patients from the AHOD with the NHR and were able to obtain the best available information for date of first positive HIV diagnosis and corresponding CD4 cell count for each participant in AHOD. We calculated the proportion of patients diagnosed with advanced HIV in AHOD (20%), which compares directly with the estimate provided yearly from the NHR (18–19%) [9]. Factors associated with increased odds of late diagnosis with advanced HIV correlate with previously published estimates. Several Australian studies have shown that sex, age, non-MSM modes of HIV exposure, country of birth and regional setting are associated with increased odds of advanced HIV diagnosis [17–20]. Comparing our findings to similar epidemics concentrated in men who have sex with men (MSM), we found notable similarities of risk factors for advanced HIV presentation included sex, age and overseas country of birth [21–25].

Evaluating factors associated with increased risk of advanced HIV diagnosis is important, especially in Australia where the proportion of late presenters has remained stable over time. In Australia, the estimate of persons infected with undiagnosed HIV are 5000–11,500 [9,26]. The proportion of new diagnoses likely attributed to transmission from undiagnosed HIV persons is estimated to be 31% [12]. Our data corroborate and support the strategy to further scale up targeted HIV testing (point-of-care or standard) towards appropriate at-risk populations, including those born or who frequently visit high prevalent HIV countries, MSMs attending sexual health clinics in non-state capital city settings and older aged MSM populations [27].

Of those AHOD participants diagnosed with advanced HIV and regardless of year of diagnosis, the majority of patients initiated antiretroviral therapy within three months of a first positive HIV diagnosis. This finding is consistent with results published from other similar international cohort studies [23,28–30]. Time to ART initiation within this group is unlikely to get any shorter. It is has long been established that initiating ART in immune-deplete patients as soon as possible reduces AIDS-related illness and mortality. Considerations for the delaying the timing ART in those that present with advanced HIV are largely based on individual circumstances. Delaying the initiation of ART within this group might be in part due to the management of prior comorbidities that might complicate ART or negatively influence adherence (e.g. mycobacterium tuberculosis) [31,32].

Evaluating the time from HIV diagnosis to time to antiretroviral therapy initiation in non-advanced HIV diagnoses, our results characterize broad changes. The management strategies of initiating asymptomatic HIV-positive persons on treatment have evolved over the time and this is reflected in our data. In recent times, the so-called treatment pendulum has swung back towards ART initiation rates comparable to the “hit hard, hit early” treatment era [1]. Earlier treatment initiation is partly gaining momentum due to better available antiretroviral formulations and reassuring results from longer term ART exposure follow-up studies [33]. In addition, evidence supporting suppressive ART reducing onwards transmission risk at both an individual and population level is growing. In Australia it is now recommended to consider ART initiation in asymptomatic patients regardless of CD4 cell count [34]. Our data indicate that initiation of ART based on a CD4 cell count threshold has been followed accordingly throughout AHOD's follow-up. These data should be informative for future evaluations of rates of ART initiation, especially as treatment-as-prevention strategies are adopted and implemented in Australia [35].

The hazard of switching a class of antiretroviral from the initial ART regimen is similar by HIV presentation groups in recent time periods. This result suggests that regardless of CD4 cell count levels at ART initiation, the durability of the first-line ART regimen is largely unaffected by the pre-treatment HIV-positive diagnosis circumstances. The rate of switching antiretroviral classes or making a major first-line ART modification presented in this analysis are similar to previous AHOD analysis [36,37] and comparable to data published from other similar cohorts [38,39]. As a measure of durability, the time to change of an antiretroviral class is preferred over evaluating any antiretroviral modification, as it is likely to correlate with a treatment failure associated first-to-second line switches [36].

In the early era of ART, treatment regimens were cumbersome and complex leading to sub-optimal adherence. The notion of a pill-break was largely considered for patients that had responded well to initial therapy and achieved higher CD4 cell counts. The Strategies for Management of Antiretroviral Therapy (SMART) trial was designed to evaluate the safety of structured treatment interruptions [40]. The trial was terminated early due to patient safety concerns. It was concluded that treatment interruptions increased the risk of mortality, new AIDS- and non-AIDS-related illnesses. In the pre-SMART era treatment interruptions were observed in approximately one-in-ten patients per year. Importantly, in recent time periods, our data show that treatment interruptions are now relatively uncommon (approximately 80% reduction). Of those participants that did interrupt therapy, our data shows a two-fold higher rate of treatment interruptions in those who initiated treatment with a higher CD4 cell count >350 cells/µL. This result likely corresponds to interruptions being largely considered for patients with higher CD4 cells counts and is consistent with other published data, where higher CD4 cell count is associated with an increased risk/rate of treatment interruption in the Asia-Pacific region [41].

There are limitations to our analysis. First, of the 3603 AHOD patient records submitted for linkage with the NHR, only 83% were matched exactly to records from the database. Theoretically all AHOD participants should have a corresponding record in the NHR as a positive HIV diagnosis is a notifiable event to health authorities. Non-matched records are likely due to the sensitivity of the data linkage key, which in this case included the combination of 2×2 name code, date of birth, sex and state/territory information. We speculate that the majority of the non-matched cases are due to incorrect 2×2 name codes supplied to AHOD or the NHR. The 2×2 name code is based on the participants first and last name at the time of diagnosis or cohort enrolment. This identifier might have changed or was incorrectly recorded at the time of notification. In addition, the true name code might not have been supplied due to the patients concern of disclosing HIV status to government authorities. The data linkage could be improved by utilizing fuzzy matching and or probabilistic joining methods. However, the lack of unique patient identifiers in AHOD and NHR, might only incrementally improve the results. Further exploration of these methods should be considered for future 2×2 name code linkage projects.

Second, of the AHOD participants matched to records from the NHR, *n*=2982, 60% were excluded from the analysis due to being diagnosed before the ART-era (*n*=1383). The exclusion criterion was imposed to reduce survivor bias in the time to ART initiation analysis. This was especially important for those diagnosed with advanced HIV. By restricting data to the ART-era only, we selected a group of participants where within each calendar year grouping, the same set of treatment options were available to physicians at the time of diagnosis. Selecting this population means that we have to rely on retrospective treatment records for those that were diagnosed (and initiated treatment) during 1996–1998. While this sub-population is small relative to the rest of the analysis cohort, we were unable to validate the completeness of their treatment records.

Finally, it is likely that our selected analysis population group is not fully representative of the entire HIV-positive population in Australia. We believe that our data is largely representative of HIV-positive persons receiving routine care. However, we acknowledge that there might be selection-bias towards persons who were not diagnosed with advanced HIV. Our results are conditional on surviving after HIV diagnosis long enough to be offered participation in AHOD. AHOD requires informed consent and it is very likely that HIV-positive persons who are diagnosed with advanced HIV are not considered for enrolment due to rapid disease progression or death.

## Conclusions

Recent HIV diagnoses are initiating therapy earlier. We note a dramatic reduction in treatment interruptions and demonstrate a stable rate of major first-line ART modifications in AHOD. In the future as treatment-as-prevention strategies scale up, more HIV diagnoses will be made across all levels of immunosuppression. The data presented provide a good indication that in recent times, HIV presentation status, and by extension CD4 cell count at ART initiation, does not largely influence the risk of treatment interruption or modification. This is reassuring as both phenomena are essential for achieving and maintaining low levels of community viral load.

## Supplementary Material

Temporal trends of time to antiretroviral treatment initiation, interruption and modification: examination of patients diagnosed with advanced HIV in Australia

## References

[1] Vella SS, Schwartländer BB, Sow SPS, Eholie SPS, Murphy RLR (2012). The history of antiretroviral therapy and of its implementation in resource-limited areas of the world. AIDS.

[2] Anglemyer AT, Rutherford GW, Easterbrook PJ, Horvath T, Vitória M, Jan M (2014). Early initiation of antiretroviral therapy in HIV-infected adults and adolescents: a systematic review. AIDS.

[3] Cohen MS, Chen YQ, McCauley M, Gamble T, Hosseinipour MC, Kumarasamy N (2011). Prevention of HIV-1 infection with early antiretroviral therapy. N Engl J Med.

[4] Tanser F, Bärnighausen T, Grapsa E, Zaidi J, Newell M-L (2013). High coverage of ART associated with decline in risk of HIV acquisition in rural KwaZulu-Natal, South Africa. Science.

[5] Granich R, Crowley S, Vitoria M, Smyth C, Kahn JG, Bennett R (2010). Highly active antiretroviral treatment as prevention of HIV transmission: review of scientific evidence and update. Curr Opin HIV AIDS.

[6] Cohen MS (2010). HIV treatment as prevention and “the Swiss statement”: in for a dime, in for a dollar?. Clin Infect Dis.

[7] Rodger A, Bruun T, Weait M, Vernazza P, Collins S, Estrada V (2012). Partners of people on ART – a new evaluation of the risks (the PARTNER study): design and methods. BMC Public Health.

[8] Rodger A, Bruun T, Cambiano V, Lundgren J, Vernazza P, Collins S (2014). HIV transmission risk through condomless sex if the HIV+ partner on suppressive ART: PARTNER study.

[9] The Kirby Institute (2013). HIV, viral hepatitis and sexually transmissible infections in Australia. Annual surveillance report 2013.

[10] Antinori A, Coenen T, Costagiola D, Dedes N, Ellefson M, Gatell J (2011). Late presentation of HIV infection: a consensus definition. HIV Med.

[11] Poli G, Pantaleo G, Fauci AS (1993). Immunopathogenesis of human immunodeficiency virus infection. Clin Infect Dis.

[12] Wilson DP, Hoare A, Regan DG, Law MG (2009). Importance of promoting HIV testing for preventing secondary transmissions: modelling the Australian HIV epidemic among men who have sex with men. Sex Health.

[13] Australian HIV Observational Database (2002). Rates of combination antiretroviral treatment change in Australia, 1997–2000. HIV Med.

[14] Petoumenos K (2003). Australian HIV Observational Database. The role of observational data in monitoring trends in antiretroviral treatment and HIV disease stage: results from the Australian HIV observational database. J Clin Virol.

[15] Mcdonald AM, Crofts N, Blumer CE, Gertig DM, Patten JJ, Roberts M (1994). The pattern of diagnosed HIV-infection in Australia, 1984–1992. AIDS.

[16] Hoy J, Lewin S (2009). HIV management in Australasia: a guide for clinical care, Darlinghurst: Australasian Society for HIV Medicine.

[17] McDonald AM, Li Y, Dore GJ, Ree H, Kaldor JM (2003). Late HIV presentation among AIDS cases in Australia, 1992–2001. Aust N Z J Public Health.

[18] Hocking JS, Rodger AJ, Rhodes DG, Crofts N (2000). Late presentation of HIV infection associated with prolonged survival following AIDS diagnosis – characteristics of individuals. Int J STD AIDS.

[19] Lemoh C, Guy R, Yohannes K, Lewis J, Street A, Biggs B (2009). Delayed diagnosis of HIV infection in Victoria 1994 to 2006. Sex Health.

[20] Wand H, Guy R, Law M, Wilson DP, Maher L (2013). High rates of late HIV diagnosis among people who inject drugs compared to men who have sex with men and heterosexual men and women in Australia. AIDS Behav.

[21] Sullivan AK, Curtis H, Sabin CA, Johnson MA (2005). Newly diagnosed HIV infections: review in UK and Ireland. BMJ.

[22] Schwarcz S, Hsu L, Dilley JW, Loeb L, Nelson K, Boyd S (2006). Late diagnosis of HIV infection: trends, prevalence, and characteristics of persons whose HIV diagnosis occurred within 12 months of developing AIDS. J Acquir Immune Defic Syndr.

[23] Sabin C, Smith C, Gumley H, Murphy G, Lampe F, Phillips A (2004). Late presenters in the era of highly active antiretroviral therapy: uptake of and responses to antiretroviral therapy. AIDS.

[24] Mugavero MJ, Castellano C, Edelman D, Hicks C (2007). Late diagnosis of HIV infection: the role of age and sex. Am J Med.

[25] Mocroft A, Lundgren JD, Sabin ML, Monforte AD, Brockmeyer N, Casabona J (2013). Risk factors and outcomes for late presentation for HIV-positive persons in Europe: results from the Collaboration of Observational HIV Epidemiological Research Europe Study (COHERE). PLoS Med.

[26] Pedrana AE, Hellard ME, Wilson K, Guy R, Stoové M (2012). High rates of undiagnosed HIV infections in a community sample of gay men in Melbourne, Australia. J Acquir Immune Defic Syndr.

[27] National HIV Testing Policy Expert Reference Committee, Ministerial Advisory Committee on Blood Borne Viruses (2011). National HIV Testing Policy v1.3.

[28] Monforte AA, Cozzi-Lepri A, Girardi E, Castagna A, Mussini C, Di Giambenedetto S (2011). Late presenters in new HIV diagnoses from an Italian cohort of HIV-infected patients: prevalence and clinical outcome. Antivir Ther.

[29] Mussini C, Manzardo C, Johnson M, Monforte AD, Uberti-Foppa C, Antinori A (2008). Patients presenting with AIDS in the HAART era: a collaborative cohort analysis. AIDS.

[30] Wolbers M, Bucher HC, Furrer H, Rickenbach M, Cavassini M, Weber R (2008). Delayed diagnosis of HIV infection and late initiation of antiretroviral therapy in the Swiss HIV cohort study. HIV Med.

[31] Battegay M, Fluckiger U, Hirschel B, Furrer H (2007). Late presentation of HIV-infected individuals. Antivir Ther (Lond).

[32] Battegay M, Fehr J, Fluckiger U, Elzi L (2008). Antiretroviral therapy of late presenters with advanced HIV disease. The Journal of Antimicrobial Chemotherapy.

[33] Eron JJ, Cooper DA, Steigbigel RT, Clotet B, Gatell JM, Kumar PN (2013). Efficacy and safety of raltegravir for treatment of HIV for 5 years in the BENCHMRK studies: final results of two randomised, placebo-controlled trials. The Lancet Infectious Diseases.

[34] Australasian Society for HIV Medicine (2013). Australian commentary on the US Department of Health and Human Services (DHHS) guidelines for the use of antiretroviral agents in HIV-1 infected adults and adolescents. http://arv.ashm.org.au/.

[35] NSW Health & New South Wales Ministry of Health (2012). NSW HIV strategy 2012–2015: a new era. http://www.health.nsw.gov.au/publications/Pages/nsw-hiv-strategy-2012-15-a-new-era.aspx.

[36] Wright ST, Boyd MA, Yunihastuti E, Law M, Sirisanthana T, Hoy J (2013). Rates and factors associated with major modifications to first-line combination antiretroviral therapy: results from the Asia-pacific region. PLoS ONE.

[37] McManus H, Hoy JF, Woolley I, Boyd MA, Kelly MD, Mulhall B Recent trends in early stage response to combination antiretroviral therapy in Australia. Antivir Ther (Lond).

[38] ART-LINC of IeDEA Study Group, Keiser O, Tweya H, Boulle A, Braitstein P, Schecter M (2009). Switching to second-line antiretroviral therapy in resource-limited settings: comparison of programmes with and without viral load monitoring. AIDS.

[39] Palombi L, Marazzi MC, Guidotti G, Germano P, Buonomo E, Scarcella P (2009). Incidence and Predictors of Death, Retention, and Switch to Second-Line Regimens in Antiretroviral-Treated Patients in Sub-Saharan African Sites with Comprehensive Monitoring Availability. Clinical Infectious Diseases.

[40] Group SFMOATSS, El-Sadr WM, Lundgren JD, Neaton JD, Gordin F, Abrams D (2006). CD4+ count-guided interruption of antiretroviral treatment. N Engl J Med.

[41] Guy R, Wand H, McManus H, Vonthanak S, Woolley I, Honda M (2013). Antiretroviral treatment interruption and loss to follow-up in two HIV cohorts in Australia and Asia: implications for “test and treat” prevention strategy. AIDS Patient Care STDS.

