# Supplementary material for: Temporal trends of time to antiretroviral treatment initiation, interruption and modification: examination of patients diagnosed with advanced HIV in Australia
Source: J Int AIDS Soc. 2015 Apr 10;18(1):19463. doi: 10.7448/IAS.18.1.19463 (PMC4394156; doi:10.7448/IAS.18.1.19463)
Supplement: Temporal trends of time to antiretroviral treatment initiation, interruption and modification: examination of patients diagnosed with advanced HIV in Australia [file JIAS-18-19463-s001.pdf]

SDC Figure 1: Data linkage results. Frequency distribution of first HIV+ test result date component (day, month, year) by database (linked NHR date, pre-linkage AHOD date).

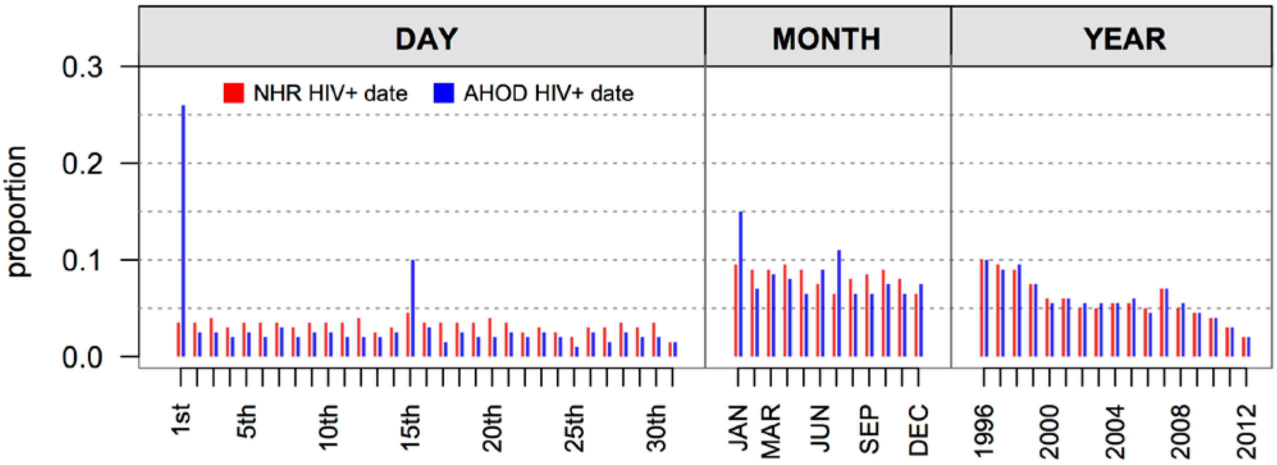

SDC Figure 2: CD4 cell count at HIV diagnosis: concordance plot between available AHOD data and corresponding NHR data (Lin's concordance correlation coefficient).

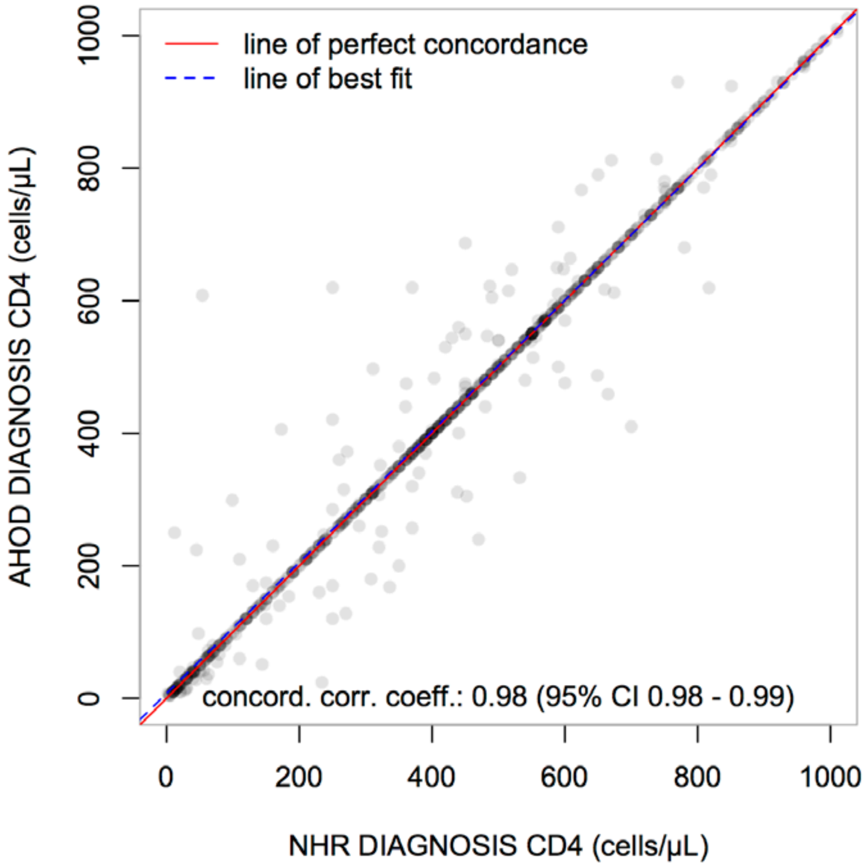

SDC Table 1: AHOD & National HIV Registry linkage results: analysis population patient characteristics by inclusion/exclusion group

Factor	Level	Overall Linkage		Matched Records	
		No Match N (%)	Matched N (%)	Included N (%)	Excluded N (%)
Number of Participants	3608	626 (17)	2982 (83)	1202 (40)	1780 (60)
Year of cohort enrolment	1999 to 2004	434 (69)	1938 (65)	497 (41)	1441 (81)
	2005 to 2009	100 (16)	613 (21)	407 (34)	206 (12)
	2010 to 2013	92 (15)	431 (14)	298 (25)	133 (7)
Sex	Female	70 (11)	157 (5)	84 (7)	73 (4)
	Male	556 (89)	2855 (95)	1118 (93)	1707 (96)
Age at cohort enrolment (years)	*less* 30	41 (7)	296 (10)	182 (15)	114 (6)
	30 to 40	232 (37)	1075 (36)	420 (35)	655 (37)
	40 to 50	218 (35)	975 (33)	359 (30)	616 (35)
	greater 50	135 (22)	636 (21)	241 (20)	395 (22)
Mode of probable HIV exposure	MSM	454 (73)	2252 (76)	842 (70)	1410 (80)
	Heterosexual	105 (17)	442 (15)	261 (22)	181 (10)
	IDU	36 (6)	184 (6)	47 (4)	137 (8)
	Other	31 (5)	104 (3)	52 (4)	52 (3)
Continental region of birth	Australia	349 (56)	1857 (62)	747 (62)	1110 (62)
	NZ/Pacific Is.	20 (3)	72 (2)	30 (3)	42 (2)
	Africa	13 (2)	39 (1)	23 (2)	16 (1)
	Americas	13 (2)	39 (1)	17 (1)	20 (1)
	Asian	39 (6)	131 (4)	78 (6)	53 (3)
	Europe	36 (6)	156 (5)	75 (6)	81 (5)
	Not reported	156 (25)	690 (23)	232 (20)	458 (26)
Hepatitis B coinfection (ever)	Positive	25 (4)	127 (4)	35 (3)	92 (5)
	Negative	498 (80)	2372 (80)	945 (79)	1427 (80)
	Not reported	103 (16)	483 (16)	222 (18)	261 (15)
Hepatitis C coinfection (ever)	Positive	76 (12)	324 (11)	84 (7)	240 (13)
	Negative	482 (77)	2351 (79)	1010 (84)	1341 (75)
	Not reported	68 (11)	307 (10)	108 (9)	199 (12)
Australian State capital city setting	Capital city	471 (75)	2106 (71)	853 (71)	1253 (70)
	Non-capital city	155 (25)	876 (29)	349 (29)	527 (30)
Primary care setting	General Practice	263 (42)	1097 (37)	467 (39)	630 (35)
	Hospital Clinic	133 (21)	664 (22)	247 (20)	417 (23)
	Sexual Health Clinic	230 (37)	1221 (41)	488 (41)	733 (41)

SDC Table 2: Factors associated with late HIV diagnosis: defined as CD4 cell count at diagnosis <350 cells/µL and or new AIDS illness within 6 months of HIV diagnosis.

			Univariate Model			Multivariable Model	
**Factor**	**Level**	**OR**	**95% CI**	**P#**	**aOR**	**95% CI**	**P#**
Sex	Female	0.96	[0.60, 1.53]	0.86	0.56	[0.32, 0.99]	0.05
	Male	1.00	[ref]		1.00	[ref]	
Age at cohort enrolment (years)	*less* 30	1.00	[ref]	<0.01	1.00	[ref]	<0.01
	30 to 40	1.32	[0.89, 1.96]		1.27	[0.84, 1.92]	
	40 to 50	1.67	[1.12, 2.49]		1.61	[1.06, 2.49]	
	greater 50	3.39	[2.23, 5.17]		2.93	[1.88, 4.60]	
Mode of probable HIV exposure	MSM	1.00	[ref]	<0.01	1.00	[ref]	<0.01
	Heterosexual	1.65	[1.24, 2.20]		1.88	[1.33, 2.67]	
	IDU	1.21	[0.65, 2.23]		1.48	[0.76, 2.91]	
	Other	2.13	[1.21, 3.74]		1.68	[0.92, 3.05]	
Continental region of birth	Australia	1.00	[ref]	<0.01	1.00	[ref]	<0.01
	NZ/Pacific Is.	0.89	[0.41, 2.02]		0.92	[0.40, 2.15]	
	Africa	3.91	[1.64, 9.35]		4.72	[1.84, 12.1]	
	Americas	2.35	[0.90, 6.16]		2.04	[0.73, 5.68]	
	Asian	1.79	[1.12, 2.86]		2.13	[1.28, 3.55]	
	Europe	1.64	[1.01, 2.65]		1.64	[0.98, 2.75]	
	Not reported	1.14	[0.84, 1.56]		0.79	[0.56, 1.12]	
Hepatitis B Co-infection	Positive	1.64	[0.84, 3.23]	0.03	1.56	[0.77, 3.18]	0.41
	Negative	1.00	[ref]		1.00	[ref]	
	Not reported	0.70	[0.51, 0.97]		0.92	[0.62, 1.37]	
Hepatitis C Co-infection	Positive	0.73	[0.45, 1.18]	0.07	0.73	[0.43, 1.24]	0.19
	Negative	1.00	[ref]		1.00	[ref]	
	Not reported	0.63	[0.40, 0.98]		0.64	[0.36, 1.11]	
Australian State capital city setting	Capital city	1.00	[ref]	0.03	1.00	[ref]	0.06
	Non-capital city	1.33	[1.03, 1.72]		1.56	[0.99, 2.46]	
Primary care setting	General Practice	0.72	[0.55, 0.94]	<0.01	0.98	[0.63, 1.54]	0.03
	Hospital Clinic	1.17	[0.86, 1.60]		1.62	[1.00, 2.61]	
	Sexual Health Clinic	1.00	[ref]		1.00	[ref]	
Year of HIV diagnosis	1996 to 2000	1.00	[ref]	<0.01	1.00	[ref]	<0.01
	2001 to 2006	0.54	[0.41, 0.72]		0.49	[0.36,0.67]	
	2007 to 2012	0.83	[0.62, 1.12]		0.77	[0.56, 1.06]	

#Wald-test for categorical level heterogeneity

SDC Table 3: Cox proportional hazard* of time to ART treatment interruption (>30 days) by HIV presentation status.

Factor	interrupt / pyrs	Rate / 100 py [95% CI]	aHR[a]	p
Year of ART initiation				
1996 to 2000	94 / 963	9.8 [8.0, 11.8]	1.00 [ref]	<0.0001
2001 to 2006	67 / 815	8.2 [6.4, 10.3]	1.07 [0.76, 1.50]	
2007 to 2012	14 / 873	1.6 [0.9, 2.7]	0.16 [0.09, 0.28]	
HIV presentation status[b]				
late HIV diagnosis	58 / 1255	4.6 [3.5, 5.9]	0.92 [0.56, 1.52]	<0.0001
$CD4_{ART}$ <350 cells/µL	24 / 596	4.0 [2.6, 5.9]	1.00 [ref]	
$CD4_{ART}$ ≥350 cells/µL	93 / 800	11.6 [9.5, 14.1]	2.00 [1.25, 3.22]	

[a]model adjusted for year of ART initiation, HIV presentation status, age, sex, HIV exposure, primary care clinic type.
[b]*late HIV diagnosis*: CD4 cell count at HIV diagnosis <350 cells/µL and or new AIDS illness within 6mth of diagnosis;
$CD4_{ART}$ <350 cells/µL: CD4 cell count at HIV diagnosis >350 cells/µL and CD4 cell count at ART initiation <350 cells/µL;
$CD4_{ART}$ ≥350 cells/µL: CD4 cell count at HIV diagnosis >350 cells/µL and CD4 cell count at ART initiation ≥350 cells/µL.

SDC Table 4: Cox proportional hazard of time to first major change to ART treatment by HIV presentation status.

Factor[b]	switch / pyrs	rate / 100 py [95% CI]	aHR[a]	p
Year of ART initiation 1996 to 2000				
late HIV diagnosis	61 / 490	12.4 [9.7, 15.7]	3.55 [1.11, 11.4]	0.04
$CD4_{ART}$ <350 cells/µL	3 / 86	3.5 [0.7, 9.8]	1.00 [ref]	
$CD4_{ART}$ ≥350 cells/µL	32 / 381	8.4 [5.8, 11.6]	2.41 [0.74, 7.88]	
Year of ART initiation 2001 to 2006				
late HIV diagnosis	22 / 371	5.9 [3.8, 8.8]	0.58 [0.32, 1.05]	0.08
$CD4_{ART}$ <350 cells/µL	22 / 203	10.8 [6.9, 15.9]	1.00 [ref]	
$CD4_{ART}$ ≥350 cells/µL	16 / 314	5.1 [2.9, 8.1]	0.51 [0.27, 0.98]	
Year of ART initiation 2007 to 2012				
late HIV diagnosis	22 / 280	7.9 [5.0, 11.7]	0.88 [0.49, 1.58]	0.21
$CD4_{ART}$ <350 cells/µL	24 / 249	9.6 [6.3, 14.0]	1.00 [ref]	
$CD4_{ART}$ ≥350 cells/µL	30 / 202	14.9 [10.2, 20.5]	1.41 [0.82, 2.41]	

[a]model adjusted for year of ART initiation, HIV presentation status, age, sex, HIV exposure, primary care clinic type.
[b]*Late HIV diagnosis*: CD4 cell count at HIV diagnosis <350 cells/µL and or new AIDS illness within 6mth of diagnosis;
$CD4_{ART}$ <350 cells/µL: CD4 cell count at HIV diagnosis >350 cells/µL and CD4 cell count at ART initiation <350 cells/µL;
$CD4_{ART}$ ≥350 cells/µL: CD4 cell count at HIV diagnosis >350 cells/µL and CD4 cell count at ART initiation ≥350 cells/µL..
